# Botulinum Neurotoxin Detection Methods for Public Health Response and Surveillance

**DOI:** 10.3389/fbioe.2018.00080

**Published:** 2018-06-22

**Authors:** Nagarajan Thirunavukkarasu, Eric Johnson, Segaran Pillai, David Hodge, Larry Stanker, Travis Wentz, BalRam Singh, Kodumudi Venkateswaran, Patrick McNutt, Michael Adler, Eric Brown, Thomas Hammack, Donald Burr, Shashi Sharma

**Affiliations:** ^1^Division of Microbiology, Office of Regulatory Science, Center for Food Safety and Applied Nutrition, U.S. Food and Drug Administration, College Park, MD, United States; ^2^Department of Bacteriology, University of Wisconsin-Madison, Madison, WI, United States; ^3^U.S. Food and Drug Administration Office of Laboratory Science and Safety, Silver Spring, MD, United States; ^4^Chemical and Biological Defense Division, Science and Technology Directorate, U.S. Department of Homeland Security, Washington, DC, United States; ^5^U.S. Department of Agriculture, Agriculture Research Service Albany, Albany, CA, United States; ^6^Institute of Advanced Sciences, Botulinum Research Center, Dartmouth, MA, United States; ^7^Omni Array Biotechnology, Rockville, MD, United States; ^8^United States Army Medical Research Institute of Chemical Defense, Aberdeen, MD, United States; ^9^Office of Regulatory Affairs, Center for Food Safety and Applied Nutrition, U.S. Food and Drug Administration, Silver Spring, MD, United States

**Keywords:** botulinum neurotoxin, *Clostridium botulinum*, botulism, public health, biosurveillance, biosecurity

## Abstract

Botulism outbreak due to consumption of food contaminated with botulinum neurotoxins (BoNTs) is a public health emergency. The threat of bioterrorism through deliberate distribution in food sources and/or aerosolization of BoNTs raises global public health and security concerns due to the potential for high mortality and morbidity. Rapid and reliable detection methods are necessary to support clinical diagnosis and surveillance for identifying the source of contamination, performing epidemiological analysis of the outbreak, preventing and responding to botulism outbreaks. This review considers the applicability of various BoNT detection methods and examines their fitness-for-purpose in safeguarding the public health and security goals.

## Introduction

Botulinum neurotoxins (BoNTs) are metalloendoproteases produced as protein complexes by anaerobic spore forming neurotoxigenic clostridia (*Clostridium botulinum, C. argentinense, C. butyricum* and *C. baratii* spp). Among the seven known BoNTs serotypes (serotype A–G), type A, B, E, and F are commonly linked to cause food-borne botulism in humans, while type C and D are commonly associated with botulism in animals and birds. BoNTS are the most poisonous substances known (Pourshaban et al., 2002), and more than 40 subtypes of BoNTs have been described based on amino acid sequence differences (Peck et al., 2017). Botulism is characterized by flaccid muscle paralysis due to inhibition of neurotransmitter release at nerve-muscle junctions (NMJ). BoNTs internalize peripheral nerve terminals of NMJ and specifically cleave Soluble *N-*ethylmaleimide-sensitive factor attachment protein receptor (SNARE) proteins, crucial for the synaptic vesicle membrane fusion and neurotransmitter release during neuroexocytosis. The tridomain structural organization and the trimodular function of BoNTs contribute to three major steps of BoNT intoxication process: receptor-mediated endocytosis, membrane translocation across the endosomes, and inhibition of acetylcholine release by cleaving SNARE proteins (Rossetto et al., 2014).

The most common form of botulism is food-borne. Neurotoxigenic clostridia can grow and form BoNTs in diverse food sources due to improper processing and subsequent temperature abuse complemented with anaerobic condition during storage or distribution. Consumption of food containing preformed toxin cause food-borne botulism, and quantities as low as 30 ng can cause food-borne botulism (Peck, 2006). Other notable forms of botulism are, (i) infant botulism, caused as a result of colonization of *C. botulinum* spores in infant gastrointestinal tract, and (ii) wound-borne botulism, in which spores grow to produce and release of toxin within injured tissues (Lindstrom and Korkeala, 2006). Adult intestinal toxemia can occur in rare cases due to similar colonization in intestine after prolonged antibiotic usage or bowel surgery (Sheppard et al., 2012), while iatrogenic botulism can occur due to accidental overdose during therapeutic or cosmetic application of BoNTs. Animal experiments indicate BoNTs also exert inhalational toxicity, and the deliberate contamination of food sources and/or the aerosolizing of BoNTs are substantial security and global public health concerns (Arnon, 2001).

Food contaminated by BoNTs can lead to large outbreaks of botulism, resulting in potentially high morbidity and mortality rates. Therefore, a single suspected case of food-borne botulism is sufficient to evoke a public health emergency. Many sporadic botulism outbreaks involve home-canned, cured, or fermented foods (Lindstrom and Korkeala, 2006), which are limited in scope to a family or small socially-connected group of people. When additional suspected cases are clustered to the same source or vehicle, outbreaks are no longer considered “sporadic” (St Louis et al., 1988). Estimating the scale of outbreaks has proven difficult in the past and it is not unusual for additional cases to come to light after an investigation has begun, and some outbreaks are only confirmed after reassessment following additional morbidity and mortality (Centers for Disease Control Prevention, 1998). In the United States, botulism outbreaks are investigated and responded by U.S. Centers for Disease Control and Prevention (CDC) to provide active clinical surveillance. Additionally, the U.S. Food and Drug Administration (FDA) and the U.S. Department of Agriculture (USDA) investigates botulism outbreaks linked to commercial food products for regulatory actions, and to determine product tampering, intentional or unintentional contamination. The Laboratory Response Network (LRN) and the Food Emergency Response Network (FERN) laboratories composed of local, state and federal public health laboratories provides response to public health emergencies. Presently, there is no permissible level of BoNTs in foods to form a basis for risk management system, due to severity of the illness (Anderson et al., 2011). Increased food production on much larger scales, innovations in food product and process development combined with supply chain modernization has amplified the risks for large scale outbreaks of botulism due to accidental or intentional contamination of a centralized source. Hence, the Food Safety Modernization Act (FSMA) mandates strategies to strengthen the entire food safety system and prioritize the prevention of outbreaks before they occur. Some ways to accomplish this involves the development of guidelines and tools that mitigate contamination hazards within the food supply.

**Figure 1:**
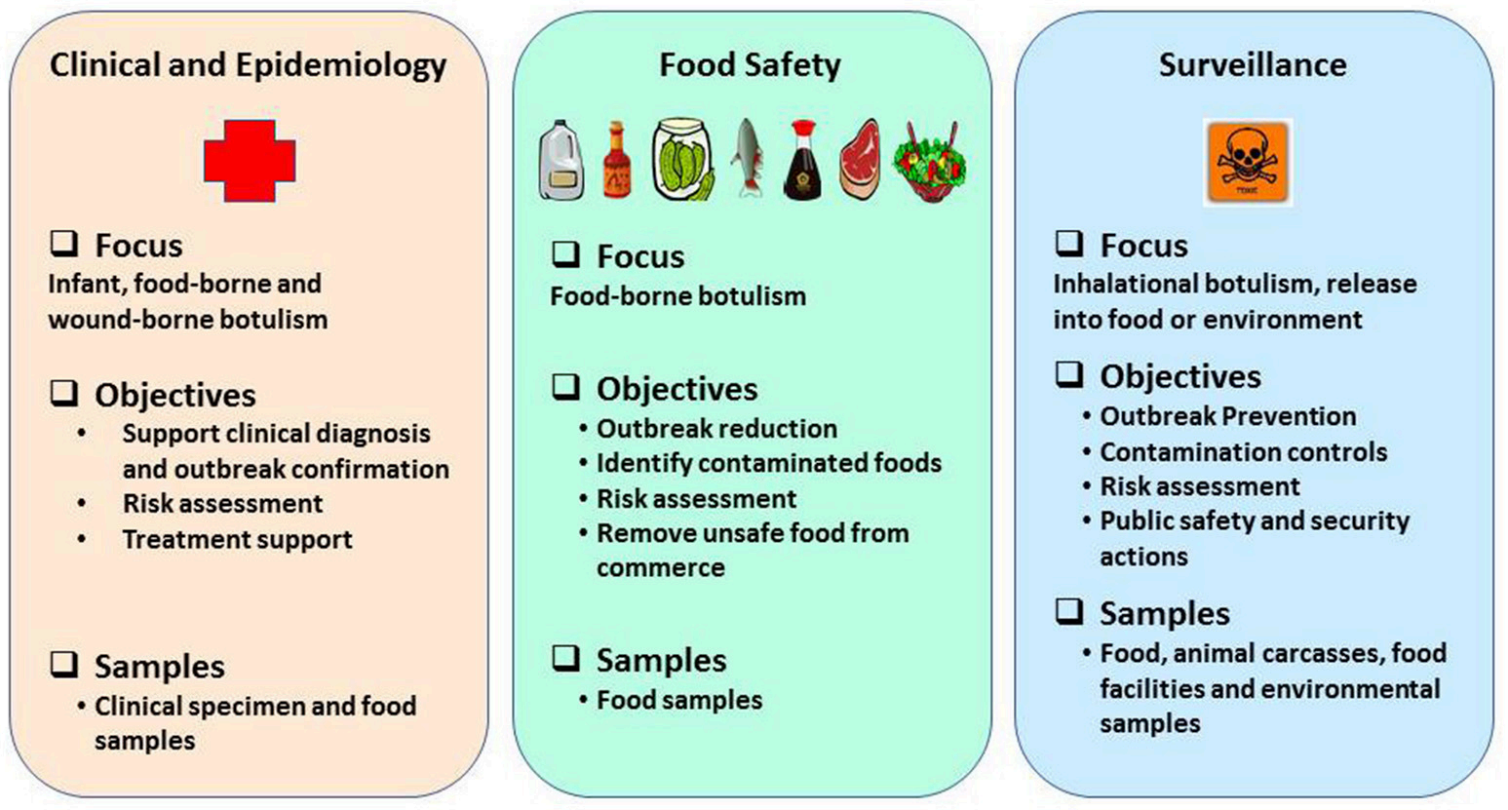
Schematic representation of public health mission goals to prevent and respond to botulism outbreaks.

Rapid and robust detection methods and precise tools are warranted to support surveillance, quickly recognize outbreaks and leverage response frameworks in a timely manner (Maslanka et al., 2016) and they must be based on emerging knowledge about BoNT subtypes or sequence types, toxin hybrids, and ability to detect mixed serotyped produced by multivalent strains of *C. botulinum* (Barash and Arnon, 2014; Fan et al., 2016; Maslanka et al., 2016). Reliable detection assays and methods are a prerequisite to prevent and respond in support of biodefense and public health missions in a timely manner. Laboratory methods to detect BoNTs or neurotoxigenic clostridia must provide timely and robust support in three major categories: (1) clinical and epidemiological investigations after an outbreak is suspected; (2) source tracking to facilitate regulatory decisions and enforcement actions to reduce likelihood of larger outbreaks, and (3) risk based/prevention-focused toxin surveillance. The overall public health mission goals and objectives to prevent and respond to botulism outbreaks are schematically represented as shown in the Figure 1. This review highlights suitability of various detection methods for preventing and responding to botulism outbreaks in the framework of mission goals to safeguard public health and security.

## Clinical diagnosis and treatment

Food-borne botulism is initially suspected based on the clinical case presentation of the patient. Cases of botulism are confirmed through laboratory identification of the direct presence of BoNTs and/or those clostridia that produce BoNTs in clinical specimens, or by the presence of BoNTs in suspect food sources consumed by the patient (Cheng et al., 2012). Symptoms typically appear within 12–36 h after toxin ingestion, although symptoms have been observed as early as 6 h or as late as 10 days after exposure (Centers for Disease Control Prevention, 1998). It is often a challenge for physicians to recognize the early symptoms of botulism due to the rarity and often sporadic nature of most outbreaks (Anderson et al., 2011). The initial symptoms of botulism can be gastrointestinal (constipation, abdominal pain, nausea, vomiting and diarrhea) which may be of parasympathetic origin (Adler and Franz, 2016). But it could also be due to ingestion of clostridial products or substances resulting from anaerobic spoilage unrelated to *C. botulinum*, which can delay recognition of the illness as botulism and early intervention. However, as neurological involvement begins to manifest, patient(s) exhibit dryness of mouth, double vision, blurred vision, drooping of upper eyelids, difficulty in swallowing and speaking, dysarthria and generalized weakness. This cluster of signs and symptoms allows physicians to distinguish cases of botulism from more common conditions often seen with other foodborne illnesses. Patients with severe cases of botulism exhibit bilateral flaccid paralysis of arms and legs and difficulty in breathing, due to paralysis of the respiratory muscles (Centers for Disease Control Prevention, 1998). Nonetheless, physicians treating these patients must still perform proper differential diagnosis to exclude other neurologic disorders such as Guillain-Barre' syndrome, myasthenia gravis, cerebrovascular accident (CVA), tick paralysis, stroke, poliomyelitis and other forms of bacterial, fungal, and chemical food poisoning which can produce similar signs and symptoms (Cai et al., 2007). Recognizing inhalational botulism by physicians can be very challenging. Limited data available from the cases who had accidental exposure to BoNTs through inhalational route and experimental studies involving primates, indicate that the clinical symptoms and the latent period of inhalation botulism are comparable to food-borne or other form of human botulism (Adler and Franz, 2016). Sudden incidence of large number of cases, unusual clustering or distribution of cases, cases lacking history for the presence of obvious wounds, clinical or cosmetic use, common dietary exposure can represent aerosolized or bioterrorism attack using BoNTs (Martin and Adams, 2003). Civilian attack involving purified toxin preparations are unlikely to produce gastrointestinal symptoms (abdominal pain, nausea, vomiting, and diarrhea), which are more typically seen in cases who have consumed food with concomitant spoilage (Adler and Franz, 2016).

Immediate administration of BoNT antitoxin along with supportive therapy is the only available treatment to stop the progression of early clinical symptoms, prevent the onset of paralysis, and accelerate recovery from paralysis (Centers for Disease Control Prevention, 1998). Hence, the decision to initiate treatment is entirely based on clinical presentation and diagnosis by the physician, rather than contingent upon laboratory investigation and confirmation, which can take several days (Singh et al., 2013). Although early administration of antitoxin effectively neutralizes and clears the toxin from blood circulation, once the BoNTs enter the nerve terminals, antitoxin is not effective for preventing the progression of botulism and does not promote early recovery from paralysis (Tacket et al., 1984). Patients with botulism may require ventilator support for 2–8 weeks, although in severe cases patients may require several months of such support (Centers for Disease Control Prevention, 1998). Without timely medical intervention, the case-fatality ratio for botulism can be as high as 60%, as reported during the years 1899–1949, before antitoxin therapy was widely available (Centers for Disease Control Prevention, 1998). Availability of antitoxins and improvements in supportive and intensive respiratory care has dramatically reduced the fatality rate to 5–10% (Centers for Disease Control Prevention, 1998; Cheng et al., 2016). Currently, there are no alternative post-exposure therapies available to treat botulism (Capek and Dickerson, 2010; Bremer et al., 2017).

## Public health response, food and biothreat surveillance

### Laboratory and epidemiological investigations

During botulism investigations public health laboratories routinely test clinical specimens (serum, feces, gastric aspirates, or vomitus) and/or suspected food sources (Centers for Disease Control Prevention, 1998) for the presence of BoNT or clostridial strains that produce BoNTs. Laboratory investigation to confirm inhalational botulism or bioterrorism would involve testing BoNT in blood, nasal mucosa, environmental samples and blood specimen with goals also to detect uncommon BoNT serotypes (type C, D, and G) that are not known to cause human botulism in humans or toxin variants. Quantitation of BoNTs in clinical specimen is not essential (Kalb et al., 2015a), although wealth of such data might help to potentially correlate the human toxic doses to classify morbidity levels from mild to severe or fatal cases of botulism. However, quantity determination in food sources also helps accurate risk assessments. Isolation of neurotoxigenic clostridia from consumed food samples do not provide confirmatory evidence of an outbreak without additional laboratory testing for the presence of toxin (Centers for Disease Control Prevention, 1998). However, instances of suspected infant botulism or wound botulism are often established by confirming the presence of neurotoxigenic clostridia in stool or gastric aspirates or in wound debris, respectively (Kalb et al., 2015a).

*In vivo* mouse lethality bioassay (MLB) is routinely used standard method to detect and confirm the presence of BoNTs in clinical specimen and food or environmental samples linked to botulism outbreaks. One of the benefits of the MLB is also that it holistically represents the trimodular domain function, and demonstrates the biological activity of BoNTs to cause botulism. This includes simulating the symptomatic progression of (untreated) human botulism, as they lead to paralysis and death, using mice (Lindstrom and Korkeala, 2006; Capek and Dickerson, 2010). MLB also possess the ability to detect diverse spectrum of BoNT subtypes described in the literature (Peck et al., 2017). Depending upon the availability of samples suspected of containing BoNTs, identification of the toxin serotype associated with the outbreak is achieved through cross-neutralization test in mice using antibodies specific to serotypes (A–G) (Cheng et al., 2016). Antitoxin neutralization assay to identify the toxin serotype/subtypes are largely used for epidemiological surveillance of the disease and meta-analyses of outbreaks. Neutralization assay also provides greater confidence to support clinical diagnosis and in confirming botulism outbreak, since food samples can contain wide range of adulterants or irrelevant toxins which can subdue or skew the observational endpoints of botulism in MLB. MLB can also be interfered due to presence of proteases, low-molecular weight compounds, or due to presence of other bacteria present in the specimen (Horwitz et al., 1976; Dezfulian and Bartlett, 1985). Performing the antibody neutralization assay with monovalent diagnostic antisera can also reveal the involvement of more than one BoNT serotype or novel serotype/toxin-variants in an outbreak (Barash and Arnon, 2014; Fan et al., 2016; Maslanka et al., 2016).

The MLB, however, suffers from several limitations. It is cost and time intensive method, as it requires an animal facility, skilled and dedicated personnel, and can take 1–4 days to complete the testing. The need for number of mice to test high volume of samples during outbreak investigations and the necessity to demonstrate the death of mice through respiratory impairment for confirming the toxicity of BoNTs, raises ethical concerns (Adler et al., 2010; Wilder-Kofie et al., 2011; Cheng et al., 2012; Sesardic, 2013). Moreover, the MLB is not always a failure-proof method for detecting the presence of BoNT in clinical specimens. For example, in a type-A botulism outbreak involving 28 cases, toxin was detected only in the serum or stool specimens of 14 individuals (MacDonald et al., 1985). Laboratory analyses could not demonstrate the presence of toxin in clinical specimen or in food samples in another outbreak involving 4 unrelated individuals who had been clinically diagnosed as having botulism after dining in the same restaurant (Centers for Disease Control Prevention, 1998). In a larger outbreak involving 59 individuals, laboratory analysis confirmed the presence of type B toxin only in a subset of patients (Terranova et al., 1978). Analysis of 309 adult cases of botulism from the outbreaks between the years 1975 and 1988 revealed that only 40–44% of serum and stool specimens obtained within 3 days of BoNT exposure were tested positive for the presence of toxin. Only 15–23% of specimens were tested positive for serum and stool specimen that were obtained after 3 days (Woodruff et al., 1992). Overall, only 65% of patients who had been clinically diagnosed with botulism could have their diagnosis confirmed by at least one laboratory method (Woodruff et al., 1992; Centers for Disease Control Prevention, 1998). In another report involving a clinical review of infant botulism cases, 3 of the 20 patients had negative tests with the standard MLB, while they showed complete recovery following treatment with BIG-IV® or BabyBIG® antitoxin treatment. Interestingly, an *in vitro* PCR assay tested positive for type B botulism (Khouri et al., 2017). Although the amount of toxin consumed (which is difficult to discern), the timing of clinical sample collection and testing by may affect the likelihood of obtaining accurate positive results, along with other contributing factors. Perhaps, this could be explained that after the entry of toxin through oral route, several physiological or innate immune factors might diminish the potency or the activity of the uninternalized toxin fraction during its resident time in physiologic system or in systemic circulation. This can decrease the probability of further detection by the MLB, although botulism was conferred in the patients. Hence, it is possible that relying on laboratory methods meant to only detect toxin activity to confirm botulism, may compromise clinical investigations and diagnosis. Nevertheless, the MLB is the currently gold-standard method in public health investigations due to its high sensitivity for detecting BoNTs and confirming the presence of biologically active form of toxin.

### Source tracking investigations and regulatory enforcement for food safety

In the United States, FDA regulates a portion of food supply, including seafood, dairy, bottled water and produce, while the USDA regulates meat, poultry and processed egg products. The basic regulatory goals for ensuring food safety are: (1) alerting the public about the food sources linked to outbreaks; (2) notifying and monitoring producers; (3) enforcing food safety regulations; (4) identifying the means by which food has become contaminated, whether on the farm, during processing, shipment, storage, or preparation to eliminate those sources of contamination; (5) enhancing regulatory tools with validated new technologies; and (6) refining the guidance for good agricultural and/or manufacturing practices (USFDA)^1^.

The goal of source tracing investigations in response to an outbreak is to identify which food sources have become contaminated by biological hazards to eliminate those foods from the distribution system or commerce, as quickly as possible. Source tracking involves linking clinical cases or clusters of illnesses, often reported from different geographic areas to a common source. Detecting the presence of toxin in food samples is a reliable approach for source-tracking, since it is the pre-formed toxin that causes an outbreak. Regulatory decisions such as recalls, seizure, injunction, administrative detention of (food) products, civil money penalties and/or prosecution and license suspensions or declarations of public health emergencies must be legitimately grounded on robust laboratory methods that can reliably confirm the presence of a definite health hazard that harm public health. Just as it is important not to underestimate the risk to public health posed by food-borne botulism outbreaks, it is also important not to cause unnecessary panic, food waste, or economic losses to the food industry or food commerce (Hussain and Dawson, 2013), in recalls, lost sales and legal expenses.

Assays that cannot confirm the presence of biologically-active form of toxin in food products, or that detect *C. botulinum* alone without any supporting laboratory data may limit regulatory enforcement actions, for the following reasons:

- C. *botulinum* spores are ubiquitously found in soils, dust, marine and freshwater sediments of wetlands, rivers, and lakes or in oxygen-free sediments or oligotrophic environments; surfaces of fruits and vegetables; in the intestinal tract of healthy fish, birds and mammals that can be potentially mobilized over to food sources; Low levels of *C. botulinum* are often present in food sources harvested from soil, water, and other environments (Anderson et al., 2011). The mere presence of *C. botulinum* in food sources does not necessarily lead to the production of BoNTs or result in illnesses. Favorable physiological conditions (pH, redox potential, ionic strength, temperature, water activity, etc) are needed to stimulate the growth of clostridia and toxin production on food substrates (Peck, 2006; Anderson et al., 2011). Specifically, nitrogenous sources were known to influence clostridial growth, as well as the production of BoNTs (Patterson-Curtis and Johnson, 1989, 1992); high levels of arginine seem to significantly repress toxin production, slow down autolysis and reduce endospore production (Fredrick et al., 2017), highlighting the significance of physiological stimuli in toxin formation.
- Existing commercial food manufacturing practices, processing methods, preservative methods, and agricultural practices can potentially destroy toxin activity. Food preservatives (nitrite, sorbic acid, parabens, phenolic antioxidants, polyphosphates and ascorbates), or pretreatments meant to reduce pathogen populations, and the presence of competing organisms (especially those produce acid resulting in lower pH), are known to inhibit the growth of *C. botulinum* and limit toxin production (Centers for Disease Control Prevention, 1998).
- Food-borne physiological conditions may also deactivate or diminish toxin activity by altering its modular or holistic functions (e.g., cell binding, internalization and enzymatic properties), and limit the ability the toxin to cause botulism outbreaks. Our understanding of the toxin (complex) formation, its stability and potency in food or environmental sources is still limited. BoNT stability and potency when produced or released during autolysis on different food substrates can vary and may depend upon the growth phase, strain that produces the toxin. They may also vary or be altered depending upon the physicochemical properties of food sources in which *C. botulinum* grow. Our present knowledge about the *in vitro* stability and potency of BoNTs, which are known to be influenced by the composition of the toxin complex containing non-toxic, accessory proteins (Rossetto et al., 2014) are derived from purified laboratory preparations or drug formulations (e.g., BOTOX®). It has been also reported that BoNTs can also be degraded by aerobic bacteria (Espelund and Klaveness, 2014). Thus, confirming the food source(s) of an outbreak would depend on the ability of the method to identify the presence of biologically active toxin. It is also possible for inactive toxins or toxin antigens to be present in some food and environmental sources as background.
- Recent reports also indicates occurrence of botulinum neurotoxin sequence homologs in non-clostridial strains of *Weissella oryzae* (Mansfield et al., 2015; Zornetta et al., 2016), *Chryseobacterium piperi* (Wentz et al., 2017), and in *Enterococcus sp* (Brunt et al., 2018). Notably, BoNT sequence homologs were also reported to cleave SNARE proteins that BoNTs target. While the health risks imposed by these toxin-homologs are not yet understood, it is possible that presence of these organisms or other cross-reactive antigens can potentially mislead laboratory detection.

In botulism investigations, the MLB is the gold standard assay for confirming the clinical diagnosis of botulism, and is also a confirmatory method for making regulatory decisions for food regulated by FDA and USDA. Results from the MLB captures the internalization potential of the toxin critical for causing botulism, provide the evidence necessary to justify enforcement actions on regulated food and food products, and it also reliably link the source of toxin contamination to the clinical cases during an outbreak investigation. Detection methods that cannot differentiate between active or inactive forms of BoNTs could potentially give false alarms and misidentify which food products or sources have been contaminated with active toxin, leading to inappropriate or inadequate response to outbreaks, tampering events, intentional contamination or bioterrorism. The MLB is also a standard method in the pharmaceutical industry to assess the potency and safety testing of BoNTs in drug products, defined by trimodular toxin's biological activity (Sesardic, 2013).

### Food, environmental, biothreat surveillance

Surveillance applications are focused on preventing an outbreak due to natural causes, or during intentional or threatened release situations. However, it is important to distinguish between types of exposure. Because, *C. botulinum* spores are ubiquitously distributed in the environment. Prevalence and persistence of certain phylogenetic groups or strains that produce specific serotypes endemic in certain soil types or geographic area are often reported (Gessler and Böhnel, 2006). Decomposition of plants, algae, and animals can create anaerobic environments that facilitate their growth and toxin production. Algae, plants, invertebrates (such as snails, earthworms, maggots, and nematodes), carcass of decaying animals, rotting plant and bio-wastes, seem to be a biotic reservoir for BoNT or *C. botulinum* which can contaminate enter into food webs or food sources to cause natural botulism outbreaks (Espelund and Klaveness, 2014; Jackson et al., 2015). Intentional release of botulinum neurotoxin could be in the form of aerosolized bioweapon to cause inhalational botulism (Arnon, 2001), or through contamination of food sources with *C. botulinum* or BoNTs (pure or crude preparations). Deliberate release of chemical and biological agents such as sarin gas, botulinum toxin (Arnon, 2001), anthrax spores, ricin, and attempts to obtain Ebola (Okumura et al., 2013) emphasize the need in our preparedness to prevent and respond to similar attacks, if carried out through the food supply or through aerosol release.

The overall goals of the detection methods in surveillance applications are to identify outbreak risks, provide situational awareness and ensure that the regulatory and public health responses are optimal and informed. Surveillance methods can be used to qualitatively evaluate presumptive presence of *C. botulinum* strains or BoNTs in samples from processing facilities and environmental sources to determine operating procedures, implement process controls for safe food product development and monitor acts of terrorism targeting the food supply or environmental sources.

## *In vitro* methods for detection of BoNTs and neurotoxigenic clostridia

A variety of methods have been described for detection of BoNTs (Singh et al., 2013; Koh et al., 2015; Babrak et al., 2016; Cheng et al., 2016), but only a small subset of these methods have undergone inter-laboratory validation of their performance criteria in different food, environmental, and clinical sample matrices, which is prerequisite for deploying any assay for use in public health laboratories. Detection criteria and requirements for various botulinum neurotoxin detection methods for different applications are represented in Table 1.

**Table 1:** Detection criteria and requirements for botulinum neurotoxin detection methods.

	**Clinical and Epidemiology**	**Food safety**		**Surveillance**
	**Laboratory confirmation**	**Source tracking (Screening)**	**Regulatory enforcement**	**Food, environmental surveillance and ecology**
Detection criteria	BoNT antigens Clostridial DNA (Toxin genes) Accessory proteins of BoNT complex BoNT producing *Clostridia* sp. Biological/Enzymatic activity	Enzymatic activity BoNT antigens BoNT producing *Clostridia* sp.	Biological Activity (Confirmatory)	BoNT antigens Clostridial DNA (Toxin genes) Accessory proteins of BoNT complex BoNT producing *Clostridia* sp. Biological/Enzymatic activity
Method fitness	Rapidity and Sensitivity Serotyping and Sub-serotyping Genome Information	Rapidity and Sensitivity High Sample Throughput Genome Information	Rapidity and Sensitivity	Rapidity and Sensitivity Sterotyping and Sub-serotyping Cost, High Sample Throughput and Automation Genome Information

### Immunoassays for detecting BoNTs

Immunoassays provide rapid, sensitive, and reproducible detection of BoNTs, giving both qualitative and quantitative information. Sensitive and reliable immunoassay platforms could be ideally used for detecting the presence of toxin antigens in in food samples for surveillance purpose or in clinical specimens alongside clinical diagnosis, to substantially reduce false negative or inconclusive results obtained from clinical specimens. Unlike the MLB, which relies on sample dilution (Stanker and Cheng, 2012), immunoassays have an excellent dynamic range of quantification (Worbs et al., 2015). Immunoassays are more cost effective, requiring fewer dedicated personnel, less instrumentation and are not skill intensive (Lindstrom and Korkeala, 2006; Cai et al., 2007; Capek and Dickerson, 2010). Furthermore, classical immunoassays like ELISA based chemistry offer the potential for high-throughput analysis. The FDA and the CDC evaluated a digoxigenin-labeled ELISA kit capable of providing laboratory confirmation for the presence of BoNT serotypes A, B, E, and F in clinical samples (Maslanka et al., 2011) and in a variety of food matrices (Ferreira et al., 2003; Sharma et al., 2006; Singh et al., 2015). The enhanced signal ELISA method (DIG-ELISA) have been successfully deployed and used in LRN, Food and Emergency Response Network (FERN) and public health laboratories for investigating food borne outbreaks, intentional contamination. ELISA-based electrochemiluminescence (ECL) assay, Luminex xMAP® multiplex assays that uses microsphere beads conjugated to antibodies, immuno-PCR (iPCR), and centrifugal microfluidic immunoassay platform (SpinDx™) have also been demonstrated to show limits of detection comparable or better than mouse bioassay in complex sample matrices (Cheng et al., 2012; Koh et al., 2015). While most of these assays uses polyclonal antibodies for enhanced signal output and to detect diverse subtypes, development of immunoassays, based on serotype- specific monoclonals have also been described. However, use of monoclonal have certain limitations: while antibodies that could capture or detect wide spectrum of BoNT subtypes are critical, monoclonal antibodies may lack affinity to certain toxin subtypes or toxin variants. Moreover, in the absence of knowing which serotype caused the outbreak, one will have to use seven different sets of antibodies.

### Assays for detecting catalytic activity of BoNTs

Another avenue to detect and identify BoNTs is by determining the catalytic activity of the endopeptidase domain. In theory, the substrate cleavage site is unique to each of the BoNT serotypes, an *in vitro* assay capable of detecting endopeptidase activity and the cleavage of specific target substrate sequence could determine which specific BoNT serotype is present. The recently developed Endopep-MS assays determines the exact location of the cleaved substrate products by mass spectrometry. The performance of these assays has been evaluated using complex matrices, including clinical samples (Kalb et al., 2006a,b; Parks et al., 2011; Bjornstad et al., 2014; Rosen et al., 2014), food samples having a wide range of acidities, viscosities, and fat levels (Kalb et al., 2015a,b), and supernatants from cultures (Wang et al., 2014). These tests have demonstrated Endopep-MS assay to be a rapid and robust method for detecting and differentiating among BoNT serotypes. Kalb et al, have developed an Endopep-MS format which includes an additional immuno-affinity enrichment step, using beads coated with serotype-specific, high-affinity monoclonal antibodies that can bind to the heavy chain of different BoNT serotypes, enabling the Endopep-MS assay to achieve a limit of detection that is similar to, or even higher than, that of the MLB (Kalb et al., 2015a). It has been shown capable of reliably identifying the toxin serotype present in food and other outbreak samples and has even demonstrated the ability to detect the presence of more than one BoNT serotype in a given sample (Kalb et al., 2015a).

Enzymatic activity of BoNTs can also be assessed by immunoassay platforms using various detection modes. Recently, researchers evaluated an Endopep-ELISA, capable of detecting 3 BoNT serotypes (A, B, and E) by measuring their enzymatic activity in an immunoassay (Simon et al., 2015). The Endopep-ELISA uses cleavage-sensitive monoclonal antibodies that bind specifically to neo-epitope(s) generated only after the cleavage of target substrates, and therefore will not bind with substrate molecules in the uncleaved state (Wictome et al., 1999; Nuss et al., 2010; Rheaume et al., 2015). Such conditional specificity amplifies the detection signal and provides superior coverage for detecting the wide spectrum of BoNT subtypes and sequence variants (Simon et al., 2015). For example, the Endopep-ELISA was capable of detecting 1 mouse median lethal dose (MLD_50_) of BoNT/B in a variety of foods, using type B-specific monoclonal antibodies for immuno-affinity capture (Wictome et al., 1999). Use of cleavage sensitive monoclonal antibodies can however limit detecting BoNT subtypes that may have unique substrate cleavage site like described for BoNT subtype F5 (Kalb et al., 2013), and F/A hybrid (Maslanka et al., 2016) which was previously reported to be a novel type H serotype (Barash and Arnon, 2014). Also with endopeptidase activity based detection methods, designing peptide-substrate sequences optimized for improved binding or enzymatic cleavage can potentially reduce or limit the detection. While optimization of substrate sequence can increase the catalytic efficiency for certain subtypes, it can also negatively impact substrate binding and catalytic characteristics of other subtypes, as could be inferred from few other studies (Henkel et al., 2009; Whitemarsh et al., 2013; Peck et al., 2017).

### Cell-based assays for detecting biological activity of BoNTs

Other alternative assays which could potentially reduce or replace the MLB have been explored, including neurogenic cell lines and stem cell-based *in vitro* assays that can detect the biological activity of BoNTs (McNutt et al., 2013; Pellett, 2013; Kiris et al., 2014). Neural stem cell assays offer comparable sensitivity to the MLB for the detecting BoNTs, can demonstrate the trimodular biological activity of BoNTs, and provide scalability to facilitate high throughput screening. However, these assays are subject to both technical and operational constraints. The cell-based assays require well-differentiated mouse or human neural cells, derived from embryonic or induced pluripotent stem cells. The time required to obtain differentiated neural cells for deployment is not significantly shorter than the time needed to run the existing MLB. Additionally, these neural cells may be sensitive to the foods or food components being tested. In one pilot study, when cultures of mouse embryonic neural stem cells were exposed to clarified milk, green bean juice, or non-alcoholic apple cider, those cells showed visible degeneration, suggesting acute cytotoxicity due to food components (Beske et al., 2016). Nonetheless, neural stem cell-based assays have been developed and approved by the FDA for use in the potency testing of BoNT-based drug products (Fernández-Salas et al., 2012; Pharma, 2015a,b), as well as for testing toxoids and antitoxin preparations for high-throughput drug screening applications (Nuss et al., 2010; Kiris et al., 2014; Beske et al., 2016; Maslanka et al., 2016). Although there is limited benefit in deploying cell-based assays for clinical or source-tracking investigations, these assays can potentially reduce the use of animals in conducting epidemiological studies.

### Nucleic acid based methods

Several nucleic acid-based methods have been developed to identify and/or quantify the presence of neurotoxigenic clostridia or spores in food, clinical (stool or wound debris) and environmental samples; and compare the phylogenetic relatedness of those outbreak strains to support epidemiological investigations. Availability of BoNT gene sequence information allows for the design of sequence specific DNA primers to detect and identify toxin serotypes/subtypes by PCR methods (Lindstrom et al., 2001; Fach et al., 2009; Peck et al., 2010). Pulsed-field gel electrophoresis (PFGE) and amplified fragment length polymorphism (AFLP) analysis have been a highly valuable tools for worldwide laboratories to genotype and compare various strains belonging to the different phylogenetic groups of *C. botulinum* (Lúquez et al., 2015). Multilocus sequence typing (MLST) and variable number tandem repeat (VNTR) analyses provide the ability to differentiate strains within a group (Smith et al., 2015). DNA microarrays were developed using whole genomic sequence information to compare the genomes of neurotoxigenic clostridia and to establish the range of toxin subtypes present in suspect foods or in clinical samples (Raphael, 2012). However, microarrays are a probe-dependent platform and they focus more on relatively common genotypes or SNP variants.

Recent advancements in next generation sequencing technologies enable researchers to explore the whole genome of clostridia with much greater resolution and can provide highly unique identifying information in the presence of appropriate metadata regarding an outbreak sample when compared to other nucleic acid methods. Whole genome sequencing (WGS) has demonstrated the high genomic diversity of toxigenic clostridia, leading to the identification of novel genotypes (Gonzalez-Escalona et al., 2014a,b; Weedmark et al., 2015; Williamson et al., 2016). SNP-based analysis of whole genomic data and core genome MLST approaches coupled with phylogenetic analysis generally provide sufficient resolution to differentiate between toxigenic clostridial strains, even those with absolute nucleotide identity at the *bont* gene sequence and botulinum toxin gene cluster (Gonzalez-Escalona et al., 2014b; Weedmark et al., 2015; Woudstra et al., 2016). However, nucleic acid methods including WGS depend on culture enrichment techniques which frequently require 5 days for successful isolation (Haim and Timothy, 1998; Cheng et al., 2016). Use of target-specific metagenomic approaches, and use of partial microbial co-enrichment combined with shotgun metagenomic sequencing can provide detection and trace-back capabilities in substantially shorter amount of time (Ottesen et al., 2016).

## Suitability of detection methods

### Clinical investigations

Rapid, reliable and sensitive assay for detecting BoNT would not only confirm clinical diagnosis, but can potentially bridge the gap in the present ways botulism is diagnosed and treated, and in particularly providing benefits to the patients that require special care, such as pediatric patients or people who are pregnant or breast feeding (Rao et al., 2016). Assays to detect BoNTs in clinical samples must be highly sensitive, as only a small fraction of ingested toxin is expected to reach the circulation (Cheng et al., 2012); sub-picomolar levels of toxin is expected to be present in clinical specimens. Endopep-Mass spectrometry assays, electrochemiluminescence (ECL) immunoassays, immuno-PCR, and enhanced chemiluminescence-based ELISA each demonstrate high levels of sensitivity, having limits of detection comparable to the MLB, and can rapidly detect active BoNTs in sera or in other clinical matrices (Chao et al., 2004; Guglielmo-Viret et al., 2005; Cheng and Stanker, 2013; Simon et al., 2015). However, it is not essential to demonstrate the presence of biologically- or enzymatically-active toxin in clinical specimens collected from a patient suspected to have botulism, simply because a patient will not exhibit the symptoms unless the toxin is active. Moreover, the possible presence of inactivated form of toxin in clinical specimens, which might not be readily detectible through the MLB or by endopeptidase-based assays, also emphasizes the need for laboratory testing to rely on immunoassays or other *in vitro* assays during botulism related outbreak investigations. Immunoassays specific for detecting toxin antigens or even accessory proteins of the toxin complex can also serve as confirmatory evidence (Simon et al., 2015). The well-established linkage between the clinical presentation and symptoms (which shows that the toxin was active at some point) in conjunction with the detection of toxin antigens, or toxin associated accessory proteins in clinical samples, should be sufficient to confirm a clinical diagnosis and/or an outbreak. Although immunoassays have often described as adjunct methods, rather than stand-alone techniques, availability of highly sensitive immunoassay platforms can potentially guide clinical diagnoses, patient management, inform epidemiological investigations and assist risk assessments without requiring the utilization of MLB or activity based assays for detecting the toxins.

### Food, environmental surveillance and epidemiological applications

Early monitoring of food production processes and packaging facilities and/or the environmental presence of *C. botulinum* spores by culture-dependent methods and DNA detection assays or for the formation of toxin facilitate surveillance and risk management to prevent the food chain or food web from *C. botulinum* contamination that lead to natural botulism outbreaks. Effective surveillance of a complex and increasingly complex and globalized food supply chain and environmental samples will require detection methods amenable for high-throughput screening and automation. During the 2001 anthrax attack investigation, first responders and public health laboratories were inundated with high volume of suspicious samples due to public fear and panic. This clearly demonstrated the importance and necessity for rapid and high throughput screening methods during intentional or threatened release situations (Ramage et al., 2016). Screening assays should be robust and designed to minimize false-negative results, since samples showing negative results are unlikely to receive further scrutiny and confirmation. Quantitation of toxin and serotype identification informs more accurate risk assessments; estimate the mortality and morbidity rates and ensure availability of countermeasures (antitoxins) in the National Strategic Stockpile. Hence, biothreat surveillance need rapid and sensitive methods for *C. botulinum* detection to reliably identify or quantify the serotype(s) in pre-processed or post-processed food, or environmental samples to inform risk assessments; support development and implementation of appropriate emergency response plans, and public safety actions (Kalb et al., 2015a). Immunoassays platforms are quite effective for surveillance applications and are also simple, cost-effective for field deployment to address biothreat concerns. Methods such as the Endopep-MS and Endopep-ELISA can perform rapid and sensitive identification of enzymatically active BoNTs and these assays can discern the likelihood of potent toxin's presence. To support regulatory decisions and bioforensic investigations due to intentional release or bioterrorism, confirmation of presumptive positive samples for the presence of biologically active toxin through an *in vivo* assay such as MLB is essential as it provide strong evidence for the presence of biologically active form of toxin with ability to cause an outbreak.

Recent advancement in whole genome sequencing technologies provide new opportunities toward investigating botulism outbreaks in substantial ways; through its ability to identify and differentiate bioagents from very closely related pathogenic species or strains at much higher resolution, and can potentially change source-tracing, surveillance, epidemiology, bioforensic investigations (for attribution related purposes). However, sufficiently large networks of outbreak data, high quality reference databases and internationally acceptable quality standards of data and metadata will be critical to build such capabilities for the continuing enhancement of food safety and public health (Sjodin et al., 2013). Whole genomic sequencing also provides valuable insight into the presence and involvement of unusual toxin variants, antimicrobial resistance, mutational bias, geographical pattern bias (geo-spatial origin of strain), horizontal genetic transfers, multiple toxin coding sequences and finding clinical links (Gonzalez-Escalona et al., 2014a). Such data can provide greater understanding regarding strain persistence or adaptability in specific environments including food sources or processing facilities, emergence and evolution of *C. botulinum* genotypes, and better insight into recurring or linked outbreak patterns to support prevention focused goals and food safety (Shapiro et al., 2012). However, detection of toxin gene(s) by nucleic acid methods do not confirm if those regions were transcribed and translated resulting in the synthesis of a toxin that directly contributes to toxicity, which often limits risk assessments, unless complemented with culturing methods and testing for toxin production.

## Conclusion and outlook

*In vitro* detection methods like ELISA, electrochemiluminescence assay, enhanced chemiluminescence based immunoassay formats, and endopeptidase activity based Endopep-MS and Endopep-ELISA methods are rapid and highly sensitive methods that can reliably detect toxin antigens and enzymatic activity of the BoNTs in complex sample matrices. Beyond demonstrating the presence of *C. botulinum* in clinical specimen, these *in vitro* methods can also provide stand-alone evidence to confirm botulism in suspected patients and are potential alternatives to reduce reliance on the MLB. The statistical gap between the number of laboratory confirmed cases and the clinically diagnosed cases that were reported over several years of time, may possibly and partly originate because of the dependence on activity based assay like MLB, although it is highly sensitive for detecting BoNTs. Tests that only measure activity of the toxin can mislead by giving negative results, if toxin activity diminish due to intervention from physiological or native immune factors, over time. However, it remains to be seen if sensitive immunoassays that can detect toxin-antigens can substantially reduce the occurrence of false negatives or inconclusive results during clinical investigations, when compared to the assays that rely on toxin activity. *In vitro* toxin detection methods are also highly valuable for screening or presumptive testing of suspected food, environmental samples, or other sample types. Detection of toxin antigens, toxin complex-associated accessory proteins or the enzymatic activity of BoNTs are good indicators for the likely presence of biologically active forms of BoNTs in tested samples. Immunoassay-based BoNT detection methods can also be adapted in to multiplex screening platforms for detecting various other biothreat toxins and pathogens and can be performed in a rapid, high-throughput screening format, amenable for automation.

Rapid changes in food production, processing, and distribution methods, including globalization of the food supply and rising public demand for foods that are “less processed” or “preservative free” contribute to scenarios in which outbreaks of foodborne illness, including botulism, could become much larger and cross international borders. Implementation of prevention-based food safety controls can help prevent major botulism outbreaks. Hence, besides exploring strategies to restrict clostridial growth in foods, better understanding of foodborne-physiological stimuli that directly induce or regulate toxin gene expression at the transcriptional level (Connan and Popoff, 2015) would allow us to explore new preservative methods for limiting toxin production in food sources and develop food safety guidelines for the industry. However, for regulatory enforcement decisions concerning food-borne botulism linked to clinical cases and biothreat surveillance applications, the MLB remain indispensable. The MLB present the strongest form of evidence for public health response, because of its innate ability to detect the biologically active form of the toxin by demonstrating its trimodular functional activity, given the possibility that inactive forms of the toxin can occur in food and environmental samples. To mitigate risk, regulatory science requires validated methods and globally harmonized detection platforms for use in bio-surveillance and outbreak investigations. Additionally, various *in vitro* assays, and next-generation sequencing platforms need to be evaluated for routine use and intended benefit. Fitness of various BoNT detection platforms in terms of standard performance characteristics needs to be validated in multiple laboratories in the framework of their ability to support public health programs, policies and procedures to assure optimal, integrated and informed decision-making processes to achieve the goals of public health, food safety and national security.

## Author contributions

NT, EJ, SP, DH, LS, and SS contributed to conception of the review article. NT wrote the first draft of the manuscript. NT, SP, DH, LS, and SS wrote sections of the manuscript. All authors contributed to manuscript revision, read and approved the submitted version.

### Conflict of interest statement

KV is the CEO of Omni Array Biotechnology, Rockville, MD, USA. BS is also the president of Prime Bio, Inc., N. Dartmouth, MA, USA. The remaining authors declare that the research was conducted in the absence of any commercial or financial relationships that could be construed as a potential conflict of interest.

## Footnotes

^1^USFDA: https://www.fda.gov/Food/GuidanceRegulation/default.htm [Online]; https://www.foodsafety.gov/poisoning/responds/index.html [Online]; https://www.cdc.gov/vitalsigns/foodsafety-2015/index.html [Online]; https://www.fda.gov/Food/default.htm [Online].

## References

[B1] Adler M. Franz D. R. (2016). CHAPTER 4 Toxicity of Botulinum Neurotoxin by Inhalation: Implications in Bioterrorism, in Aerobiology: The Toxicology of Airborne Pathogens and Toxins, eds Salem H. Katz S. A. (Cambridge, UK: The Royal Society of Chemistry), 167–185.

[B2] Adler S. Bicker G. Bigalke H. Bishop C. Blumel J. Dressler D.. (2010). The current scientific and legal status of alternative methods to the LD50 test for botulinum neurotoxin potency testing. The report and recommendations of a ZEBET Expert Meeting. Altern. Lab. Anim. 38, 315–330. 20822324 10.1177/026119291003800401

[B3] Anderson N. M. Larkin J. W. Cole M. B. Skinner G. E. Whiting R. C. Gorris L. G.. (2011). Food safety objective approach for controlling *Clostridium botulinum* growth and toxin production in commercially sterile foods. J. Food Prot. 74, 1956–1989. 10.4315/0362-028X.JFP-11-082 22054200

[B4] Arnon S. S. (2001). Botulinum toxin as a biological weapon: medical and public health management. JAMA 285:2081. 10.1001/jama.285.8.1059 11209178

[B5] Babrak L. Lin A. Stanker L. H. McGarvey J. Hnasko R. (2016). Rapid microfluidic assay for the detection of botulinum neurotoxin in animal sera. Toxins 8:E13. 10.3390/toxins8010013 26742073 PMC4728535

[B6] Barash J. R. Arnon S. S. (2014). A novel strain of *Clostridium botulinum* that produces type B and type H botulinum toxins. J. Infect. Dis. 209, 183–191. 10.1093/infdis/jit449 24106296

[B7] Beske P. H. Bradford A. B. Grynovicki J. O. Glotfelty E. J. Hoffman K. M. Hubbard K. S.. (2016). Botulinum and tetanus neurotoxin-induced blockade of synaptic transmission in networked cultures of human and rodent neurons. Toxicol. Sci. 149, 503–515. 10.1093/toxsci/kfv254 26615023 PMC4751230

[B8] Björnstad K. Tevell Åberg A. Kalb S. R. Wang D. Barr J. R. Bondesson U.. (2014). Validation of the Endopep-MS method for qualitative detection of active botulinum neurotoxins in human and chicken serum. Anal. Bioanal. Chem. 406, 7149–7161. 10.1007/s00216-014-8170-4 25228079 PMC4522911

[B9] Bremer P. T. Adler M. Phung C. H. Singh A. K. Janda K. D. (2017). Newly designed quinolinol inhibitors mitigate the effects of botulinum neurotoxin A in enzymatic, cell-based, and *ex vivo* assays. J. Med. Chem. 60, 338–348. 10.1021/acs.jmedchem.6b01393 27966961 PMC5496736

[B10] Brunt J. Carter A. T. Stringer S. C. Peck M. W. (2018). Identification of a novel botulinum neurotoxin gene cluster in *Enterococcus*. FEBS Lett. 592, 310–317. 10.1002/1873-3468.12969 29323697 PMC5838542

[B11] Cai S. Singh B. R. Sharma S. (2007). Botulism diagnostics: from clinical symptoms to *in vitro* assays. Crit. Rev. Microbiol. 33, 109–125. 10.1080/10408410701364562 17558660

[B12] Capek P. Dickerson T. J. (2010). Sensing the deadliest toxin: technologies for botulinum neurotoxin detection. Toxins 2, 24–53. 10.3390/toxins2010024 22069545 PMC3206617

[B13] Centers for Disease Control Prevention (1998). Botulism in the United States, 1899-1996. Handbook for Epidemiologists, Clinicians, and Laboratory Workers (Atlanta, GA). 1–42. Available online at: https://www.cdc.gov/botulism/pdf/bot-manual.pdf

[B14] Chao H. Y. Wang Y. C. Tang S. S. Liu H. W. (2004). A highly sensitive immuno-polymerase chain reaction assay for *Clostridium botulinum* neurotoxin type A. Toxicon 43, 27–34. 10.1016/j.toxicon.2003.10.013 15037026

[B15] Cheng L. W. Stanker L. H. (2013). Detection of botulinum neurotoxin serotypes A and B using a chemiluminescent versus electrochemiluminescent immunoassay in food and serum. J. Agric. Food Chem. 61, 755–760. 10.1021/jf3041963 23265581 PMC3598631

[B16] Cheng W. L. Land M. K. Stanker H. L. (2012). Current Methods for Detecting the Presence of Botulinum Neurotoxins in Food and Other Biological Samples, in Bioterrorism, Book Chapter 1, ed Morse S. A. (London, UK: InTech), 1–16.

[B17] Cheng W. L. Land M. K. Tam C. Brandon D. L. Stanker H. L. (2016). Technologies for Detecting Botulinum Neurotoxins in Biological and Environmental Matrices. London, UK: InTech 125–144.

[B18] Connan C. Popoff M. R. (2015). Two-component systems and toxinogenesis regulation in *Clostridium botulinum*. Res. Microbiol. 166, 332–343. 10.1016/j.resmic.2014.12.012 25592073

[B19] Dezfulian M. Bartlett J. G. (1985). Detection of *Clostridium botulinum* type B toxin in the presence of a lethal substance interfering with toxin neutralization. Diagn. Microbiol. Infect. Dis. 3, 105–112. 10.1016/0732-8893(85)90018-5 3884243

[B20] Espelund M. Klaveness D. (2014). Botulism outbreaks in natural environments–an update. Front. Microbiol. 5:287. 10.3389/fmicb.2014.00287 24966853 PMC4052663

[B21] Fach P. Micheau P. Mazuet C. Perelle S. Popoff M. (2009). Development of real-time PCR tests for detecting botulinum neurotoxins A, B, E, F producing *Clostridium botulinum*, *Clostridium baratii* and *Clostridium butyricum*. J. Appl. Microbiol. 107, 465–473. 10.1111/j.1365-2672.2009.04215.x 19291235

[B22] Fan Y. Barash J. R. Lou J. Conrad F. Marks J. D. Arnon S. S. (2016). Immunological characterization and neutralizing ability of monoclonal antibodies directed against botulinum neurotoxin type H. J. Infect. Dis. 213, 1606–1614. 10.1093/infdis/jiv770 26936913 PMC4837907

[B23] Fernández-Salas E. Wang J. Molina Y. Nelson J. B. Jacky B. P. Aoki K. R. (2012). Botulinum neurotoxin serotype a specific cell-based potency assay to replace the mouse bioassay. PLoS ONE 7:e49516. 10.1371/journal.pone.0049516 23185348 PMC3504020

[B24] Ferreira J. L. Maslanka S. Johnson E. Goodnough M. (2003). Detection of botulinal neurotoxins A, B, E, and F by amplified enzyme-linked immunosorbent assay: collaborative study. J. AOAC Int. 86, 314–331. 12723917

[B25] Fredrick C. M. Lin G. Johnson E. A. (2017). Regulation of botulinum neurotoxin synthesis and toxin complex formation by arginine and glucose in *Clostridium botulinum* ATCC 3502. Appl. Environ. Microbiol. 83:e00642–17. 10.1128/AEM.00642-17 28455330 PMC5479000

[B26] Gessler F. Böhnel H. (2006). Persistence and mobility of a *Clostridium botulinum* spore population introduced to soil with spiked compost. FEMS Microbiol. Ecol. 58, 384–393. 10.1111/j.1574-6941.2006.00183.x 17117983

[B27] Gonzalez-Escalona N. Thirunavukkarasu N. Singh A. Toro M. Brown E. W. Zink D.. (2014a). Draft genome sequence of bivalent *Clostridium botulinum* strain IBCA10-7060, encoding botulinum neurotoxin B and a new FA mosaic type. Genome Announc. 2:e01275–14. 10.1128/genomeA.01275-14 25502671 PMC4263833

[B28] Gonzalez-Escalona N. Timme R. Raphael B. H. Zink D. Sharma S. K. (2014b). Whole-genome single-nucleotide-polymorphism analysis for discrimination of *Clostridium botulinum* group I strains. Appl. Environ. Microbiol. 80, 2125–2132. 10.1128/AEM.03934-13 24463972 PMC3993156

[B29] Guglielmo-Viret V. Attrée O. Blanco-Gros V. Thullier P. (2005). Comparison of electrochemiluminescence assay and ELISA for the detection of *Clostridium botulinum* type B neurotoxin. J. Immunol. Methods 301, 164–172. 10.1016/j.jim.2005.04.003 15979637

[B30] Haim M. S. Timothy L. Jr. (1998). Bacteriological Analytical Manual, in Chapter 17 Clostridium Botulinum, 8th Edn. Available online at: https://www.fda.gov/Food/FoodScienceResearch/LaboratoryMethods/ucm070879.htm.

[B31] Henkel J. S. Jacobson M. Tepp W. Pier C. Johnson E. A. Barbieri J. T. (2009). Catalytic properties of botulinum neurotoxins subtypes A3 and A4. Biochemistry 48, 2522–2528. 10.1021/bi801686b 19256469 PMC2701208

[B32] Horwitz M. A. Hatheway C. L. Dowell V. R. (1976). Laboratory diagnosis of botulism complicated by pyridostigmine treatment of the patient. A method for selectively removing interfering substances from clinical specimens. Am. J. Clin. Pathol. 66, 737–742. 10.1093/ajcp/66.4.737 970375

[B33] Hussain M. A. Dawson C. O. (2013). Economic impact of food safety outbreaks on food businesses. Foods 2, 585–589. 10.3390/foods2040585 28239140 PMC5302274

[B34] Jackson K. A. Mahon B. E. Copeland J. Fagan R. P. (2015). Botulism mortality in the USA, 1975-2009. Botulinum J. 3, 6–17. 10.1504/TBJ.2015.078132 28603554 PMC5460764

[B35] Kalb S. R. Baudys J. Wang D. Barr J. R. (2015a). Recommended mass spectrometry-based strategies to identify botulinum neurotoxin-containing samples. Toxins 7, 1765–1778. 10.3390/toxins7051765 25996606 PMC4448173

[B36] Kalb S. R. Baudys J. Webb R. P. Wright P. Smith T. J. Smith L. A. (2013). Discovery of a novel enzymatic cleavage site for botulinum neurotoxin F5. Toxicon 68, 76–77. 10.1016/j.toxicon.2012.07.059 PMC3263758 22172278

[B37] Kalb S. R. Krilich J. C. Dykes J. K. Lúquez C. Maslanka S. E. Barr J. R. (2015b). Detection of botulinum toxins A, B, E, and F in foods by Endopep-MS. J. Agric. Food Chem. 63, 1133–1141. 10.1021/jf505482b 25578960 PMC4523457

[B38] Kalb S. R. Moura H. Boyer A. E. McWilliams L. G. Pirkle J. L. Barr J. R. (2006a). The use of Endopep-MS for the detection of botulinum toxins A B, E, and F in serum and stool samples. Anal. Biochem. 351, 84–92. 10.1016/j.ab.2006.01.027 16500606

[B39] Kalb S. R. Moura H. Boyer A. E. McWilliams L. G. Woolfitt A. R. Barr J. R. (2006b). Detection of Botulinum Neurotoxin in Biological Samples. Abstracts of Papers of the American Chemical Society.

[B40] Khouri J. M. Payne J. R. Arnon S. S. (2017). More clinical mimics of infant botulism. J. Pediatr. 193, 178–182. 10.1016/j.jpeds.2017.09.044 29229451

[B41] Kiris E. Kota K. P. Burnett J. C. Soloveva V. Kane C. D. Bavari S. (2014). Recent developments in cell-based assays and stem cell technologies for botulinum neurotoxin research and drug discovery. Expert Rev. Mol. Diagn. 14, 153–168. 10.1586/14737159.2014.867808 24450833 PMC4478076

[B42] Koh C. Y. Schaff U. Y. Piccini M. E. Stanker L. H. Cheng L. W. Ravichandran E.. (2015). Centrifugal microfluidic platform for ultrasensitive detection of botulinum toxin. Anal. Chem. 87, 922–928. 10.1021/ac504054u 25521812 PMC4303339

[B43] Lindström M. Keto R. Markkula A. Nevas M. Hielm S. Korkeala H. (2001). Multiplex PCR assay for detection and identification of *Clostridium botulinum* types A, B, E, and F in food and fecal material. Appl. Environ. Microbiol. 67, 5694–5699. 10.1128/AEM.67.12.5694-5699.2001 11722924 PMC93361

[B44] Lindström M. Korkeala H. (2006). Laboratory diagnostics of botulism. Clin. Microbiol. Rev. 19, 298–314. 10.1128/CMR.19.2.298-314.2006 16614251 PMC1471988

[B45] Lúquez C. Joseph L. A. Maslanka S. E. (2015). Molecular Subtyping of *Clostridium botulinum* by Pulsed-Field Gel Electrophoresis, in Pulse Field Gel Electrophoresis: Methods and Protocols, eds Jordan K. Dalmasso M. (New York, NY: Springer New York), 103–113. 10.1007/978-1-4939-2599-5_10 25862052

[B46] MacDonald K. L. Spengler R. F. Hatheway C. L. Hargrett N. T. Cohen M. L. (1985). Type A botulism from sauteed onions. Clinical and epidemiologic observations. JAMA 253, 1275–1278. 10.1001/jama.1985.03350330073025 3968852

[B47] Mansfield M. J. Adams J. B. Doxey A. C. (2015). Botulinum neurotoxin homologs in non-*Clostridium* species. FEBS Lett. 589, 342–348. 10.1016/j.febslet.2014.12.018 25541486

[B48] Martin C. O. Adams H. P. Jr. (2003). Neurological aspects of biological and chemical terrorism: a review for neurologists. Arch. Neurol. 60, 21–25. 10.1001/archneur.60.1.21 12533084

[B49] Maslanka S. E. Lúquez C. Dykes J. K. Tepp W. H. Pier C. L. Pellett S.. (2016). A novel botulinum neurotoxin, previously reported as serotype H, has a hybrid-like structure with regions of similarity to the structures of serotypes A and F and is neutralized with serotype A antitoxin. J. Infect. Dis. 213, 379–385. 10.1093/infdis/jiv327 26068781 PMC4704661

[B50] Maslanka S. E. Luquez C. Raphael H. B. Dykes J. K. Joseph A. L. (2011). Utility of botulinum toxin ELISA A, B, E, F kits for clinical laboratory investigations of human botulism Botulinum J. 2, 72–92. 10.1504/TBJ.2011.041817

[B51] McNutt P. Beske P. Thirunavukkarsu N. (2013). Cell-Based Assays Cell-based assays for Neurotoxin Neurotoxins Studies, in Biological Toxins and Bioterrorism: Biological Toxins and Bioterrorism, ed Gopalakrishnakone P. (Dordrecht: Springer Netherlands), 1–22.

[B52] Nuss J. E. Ruthel G. Tressler L. E. Wanner L. M. Torres-Melendez E. Hale M. L.. (2010). Development of cell-based assays to measure botulinum neurotoxin serotype A activity using cleavage-sensitive antibodies. J. Biomol. Screen. 15, 42–51. 10.1177/1087057109354779 19965805

[B53] Okumura T. Seto Y. Fuse A. (2013). Countermeasures against chemical terrorism in Japan. Forensic Sci. Int. 227, 2–6. 10.1016/j.forsciint.2012.11.008 23434376

[B54] Ottesen A. Ramachandran P. Reed E. White J. R. Hasan N. Subramanian P.. (2016). Enrichment dynamics of *Listeria monocytogenes* and the associated microbiome from naturally contaminated ice cream linked to a listeriosis outbreak. BMC Microbiol. 16:275. 10.1186/s12866-016-0894-1 27852235 PMC5112668

[B55] Parks B. A. Shearer J. D. Baudys J. Kalb S. R. Sanford D. C. Pirkle J. L.. (2011). Quantification of botulinum neurotoxin serotypes A and B from serum using mass spectrometry. Anal. Chem. 83, 9047–9053. 10.1021/ac201910q 22017298

[B56] Patterson-Curtis S. I. Johnson E. A. (1989). Regulation of neurotoxin and protease formation in *Clostridium botulinum* Okra B and Hall A by arginine. Appl. Environ. Microbiol. 55, 1544–1548. 2669631 10.1128/aem.55.6.1544-1548.1989 PMC202901

[B57] Patterson-Curtis S. I. Johnson E. A. (1992). Roles of arginine in growth of *Clostridium botulinum* Okra B. Appl. Environ. Microbiol. 58, 2334–2337. 1637170 10.1128/aem.58.7.2334-2337.1992 PMC195778

[B58] Peck M. W. (2006). *Clostridium botulinum* and the safety of minimally heated, chilled foods: an emerging issue? J. Appl. Microbiol. 101, 556–570. 10.1111/j.1365-2672.2006.02987.x 16907806

[B59] Peck M. W. Plowman J. Aldus C. F. Wyatt G. M. Izurieta W. P. Stringer S. C.. (2010). Development and application of a new method for specific and sensitive enumeration of spores of nonproteolytic *Clostridium botulinum* types B, E, and F in foods and food materials. Appl. Environ. Microbiol. 76, 6607–6614. 10.1128/AEM.01007-10 20709854 PMC2950478

[B60] Peck M. W. Smith T. J. Anniballi F. Austin J. W. Bano L. Bradshaw M.. (2017). Historical perspectives and guidelines for botulinum neurotoxin subtype nomenclature. Toxins 9:38. 10.3390/toxins9010038 28106761 PMC5308270

[B61] Pellett S. (2013). Progress in cell based assays for botulinum neurotoxin detection. Botulinum Neurotoxins 364, 257–285. 10.1007/978-3-662-45790-0_12 23239357 PMC3644986

[B62] Pharma M. (2015a). Alternative Test Method for Botulinum Neurotoxin Now Approved in Europe. Corporate News. Available online at: https://www.merz.com/blog/news/alternative-test-method-for-botulinum-neurotoxin-now-approved-in-europe/

[B63] Pharma M. (2015b). Landmark Change for Botulinum Neurotoxin: Alternative Test Method Approved in the U.S. Corporate News. Available online at: https://www.merz.com/blog/news/botulinum-neurotoxin/.

[B64] Pourshaban M. Franciosa G. Fenicia L. Aureli P. (2002). Taxonomic identity of type E botulinum toxin-producing Clostridium butyricum strains by sequencing of a short 16S rDNA region. FEMS Microbiol. Lett. 214, 119–125. 10.1111/j.1574-6968.2002.tb11334.x 12204382

[B65] Ramage J. G. Prentice K. W. DePalma L. Venkateswaran K. S. Chivukula S. Chapman C.. (2016). Comprehensive laboratory evaluation of a highly specific lateral flow assay for the presumptive identification of bacillus anthracis spores in suspicious white powders and environmental samples. Health Secur. 14, 351–365. 10.1089/hs.2016.0041 27661796 PMC5041547

[B66] Rao A. Sobel J. Meaney-Delman D. (2016). Development of CDC Clinical Guidelines for Botulism, in 53rd Interagency Botulism Research Coordinating Committee (IBRCC) Meeting. ed Maslanka S. (Decatur, GA).

[B67] Raphael B. H. (2012). Exploring genomic diversity in *Clostridium botulinum* using DNA microarrays. Botulinum J. 2, 99–108. 10.1504/TBJ.2012.050195 PMC11310924 39130517

[B68] Rhéaume C. Cai B. B. Wang J. Fernández-Salas E. Aoki K. R. Francis J.. (2015). A highly specific monoclonal antibody for botulinum neurotoxin type A-cleaved SNAP25. Toxins 7, 2354–2370. 10.3390/toxins7072354 26114335 PMC4516917

[B69] Rosen O. Feldberg L. Gura S. Zichel R. (2014). A new peptide substrate for enhanced botulinum neurotoxin type B detection by endopeptidase-liquid chromatography-tandem mass spectrometry/multiple reaction monitoring assay. Anal. Biochem. 473, 7–10. 10.1016/j.ab.2014.09.016 25277815

[B70] Rossetto O. Pirazzini M. Montecucco C. (2014). Botulinum neurotoxins: genetic, structural and mechanistic insights. Nat. Rev. Microbiol. 12, 535–549. 10.1038/nrmicro3295 24975322

[B71] Sesardic D. (2013). Reducing animal consumption for the production of botulinum neurotoxin drugs. Toxicon 68, 73–74. 10.1016/j.toxicon.2012.07.052

[B72] Shapiro B. J. Friedman J. Cordero O. X. Preheim S. P. Timberlake S. C. Szabó G.. (2012). Population genomics of early events in the ecological differentiation of bacteria. Science 336, 48–51. 10.1126/science.1218198 22491847 PMC3337212

[B73] Sharma S. K. Ferreira J. L. Eblen B. S. Whiting R. C. (2006). Detection of type A, B, E, and F *Clostridium botulinum* neurotoxins in foods by using an amplified enzyme-linked immunosorbent assay with digoxigenin-labeled antibodies. Appl. Environ. Microbiol. 72, 1231–1238. 10.1128/AEM.72.2.1231-1238.2006 16461671 PMC1392902

[B74] Sheppard Y. D. Middleton D. Whitfield Y. Tyndel F. Haider S. Spiegelman J.. (2012). Intestinal toxemia botulism in 3 adults, Ontario, Canada, 2006-2008. Emerging Infect. Dis. 18, 1–6. 10.3201/eid1801.110533 22257757 PMC3310098

[B75] Simon S. Fiebig U. Liu Y. Tierney R. Dano J. Worbs S.. (2015). Recommended immunological strategies to screen for botulinum neurotoxin-containing samples. Toxins 7, 5011–5034. 10.3390/toxins7124860 26703727 PMC4690110

[B76] Singh A. Datta S. Sachdeva A. Maslanka S. Dykes J. Skinner G.. (2015). Evaluation of an enzyme-linked immunosorbent assay (ELISA) kit for the detection of botulinum neurotoxins A, B, E, and F in selected food matrices. Health Secur. 13, 37–44. 10.1089/hs.2014.0075 25812427

[B77] Singh A. K. Stanker L. H. Sharma S. K. (2013). Botulinum neurotoxin: where are we with detection technologies? Crit. Rev. Microbiol. 39, 43–56. 10.3109/1040841X.2012.691457 22676403

[B78] Sjödin A. Broman T. Melefors Ö O. Andersson G. Rasmusson B. Knutsson R.. (2013). The need for high-quality whole-genome sequence databases in microbial forensics. Biosecur. Bioterror. 11(Suppl. 1), S78–S86. 10.1089/bsp.2013.0007 23971825

[B79] Smith T. J. Hill K. K. Raphael B. H. (2015). Historical and current perspectives on *Clostridium botulinum* diversity. Res. Microbiol. 166, 290–302. 10.1016/j.resmic.2014.09.007 25312020 PMC11302483

[B80] Stanker H. L. Cheng W. L. (2012). Monoclonal antibodies and reagents for botulinum research. Botulinum J. 2, 150–155. 10.1504/TBJ.2012.050197

[B81] St Louis M. E. Peck S. H. Bowering D. Morgan G. B. Blatherwick J. Banerjee S.. (1988). Botulism from chopped garlic: delayed recognition of a major outbreak. Ann. Intern. Med. 108, 363–368. 10.7326/0003-4819-108-3-363 3341673

[B82] Tacket C. O. Shandera W. X. Mann J. M. Hargrett N. T. Blake P. A. (1984). Equine antitoxin use and other factors that predict outcome in type A foodborne botulism. Am. J. Med. 76, 794–798. 10.1016/0002-9343(84)90988-4 6720725

[B83] Terranova W. Breman J. G. Locey R. P. Speck S. (1978). Botulism type B: epidemiologic aspects of an extensive outbreak. Am. J. Epidemiol. 108, 150–156. 10.1093/oxfordjournals.aje.a112599 707476

[B84] Wang D. Baudys J. Krilich J. Smith T. J. Barr J. R. Kalb S. R. (2014). A two-stage multiplex method for quantitative analysis of botulinum neurotoxins type A, B, E, and F by MALDI-TOF mass spectrometry. Anal. Chem. 86, 10847–10854. 10.1021/ac502948v 25285509 PMC4582765

[B85] Weedmark K. A. Mabon P. Hayden K. L. Lambert D. van Domselaar G. Austin J. W.. (2015). *Clostridium botulinum* group II isolate phylogenomic profiling using whole-genome sequence data. Appl. Environ. Microbiol. 81, 5938–5948. 10.1128/AEM.01155-15 26116673 PMC4551264

[B86] Wentz T. G. Muruvanda T. Lomonaco S. Thirunavukkarasu N. Hoffmann M. Allard M. W.. (2017). Closed genome sequence of chryseobacterium piperi strain CTM(T)/ATCC BAA-1782, a gram-negative bacterium with clostridial neurotoxin-like coding sequences. Genome Announc. 5:e01296–17. 10.1128/genomeA.01296-17 29192076 PMC5722062

[B87] Whitemarsh R. C. Tepp W. H. Bradshaw M. Lin G. Pier C. L. Scherf J. M.. (2013). Characterization of botulinum neurotoxin A subtypes 1 through 5 by investigation of activities in mice, in neuronal cell cultures, and *in vitro*. Infect. Immun. 81, 3894–3902. 10.1128/IAI.00536-13 23918782 PMC3811745

[B88] Wictome M. Newton K. Jameson K. Hallis B. Dunnigan P. Mackay E.. (1999). Development of an *in vitro* bioassay for *Clostridium botulinum* type B neurotoxin in foods that is more sensitive than the mouse bioassay. Appl. Environ. Microbiol. 65, 3787–3792. 10473376 10.1128/aem.65.9.3787-3792.1999 PMC99701

[B89] Wilder-Kofie T. D. Lúquez C. Adler M. Dykes J. K. Coleman J. D. Maslanka S. E. (2011). An alternative *in vivo* method to refine the mouse bioassay for botulinum toxin detection. Comp. Med. 61, 235–242. 21819693 PMC3123756

[B90] Williamson C. H. Sahl J. W. Smith T. J. Xie G. Foley B. T. Smith L. A.. (2016). Comparative genomic analyses reveal broad diversity in botulinum-toxin-producing Clostridia. BMC Genomics 17:180. 10.1186/s12864-016-2502-z 26939550 PMC4778365

[B91] Woodruff B. A. Griffin P. M. McCroskey L. M. Smart J. F. Wainwright R. B. Bryant R. G.. (1992). Clinical and laboratory comparison of botulism from toxin types A, B, and E in the United States, 1975-1988. J. Infect. Dis. 166, 1281–1286. 10.1093/infdis/166.6.1281 1431246

[B92] Worbs S. Fiebig U. Zeleny R. Schimmel H. Rummel A. Luginbühl W.. (2015). Qualitative and quantitative detection of botulinum neurotoxins from complex matrices: results of the first international proficiency test. Toxins 7, 4935–4966. 10.3390/toxins7124857 26703724 PMC4690107

[B93] Woudstra C. Le Marechal C. Souillard R. Bayon-Auboyer M. H. Maréchal I. Desoutter D.. (2016). New insights into the genetic diversity of *Clostridium botulinum* group III through extensive genome exploration. Front. Microbiol. 7:757. 10.3389/fmicb.2016.00757 27242769 PMC4871853

[B94] Zornetta I. Azarnia Tehran D. Arrigoni G. Anniballi F. Bano L. Leka O.. (2016). The first non *Clostridial botulinum*-like toxin cleaves VAMP within the juxtamembrane domain. Sci. Rep. 6:30257. 10.1038/srep30257 27443638 PMC4957215

